# Effects of glucose and osmotic pressure on the proliferation and cell cycle of human chorionic trophoblast cells

**DOI:** 10.1515/biol-2022-0508

**Published:** 2022-11-07

**Authors:** Zhenyu Wang, Ding Wang, Jia Chen, Tuhong Long, Caijuan Zhong, Yingtao Li

**Affiliations:** Department of Obstetrics and Gynecology, The Third Affiliated Hospital of Guangzhou Medical University, Guangzhou, 510150, China; Guangzhou Medical Centre for Critical Pregnant Women, Guangzhou, 510150, China; Guangdong Provincial Key Laboratory of Major Obstetric Diseases, Guangzhou 510150, China; Department of Obstetrics and Gynecology, Sun Yat-Sen Memorial Hospital of Sun Yat-sen University, Guangzhou 510120, China; Key Laboratory of Reproduction and Genetics of Guangdong Higher Education Institute, Guangzhou, 510150, China; Department of Obstetrics, Foshan Women and Children Hospital, Foshan 528000, China; Department of Medical Affairs Section, The Third Affiliated Hospital of Guangzhou Medical University, Guangzhou 510150, China; Department of Obstetrics, Maternal & Child Health Hospital of Guangdong, Guangzhou 510010, China; Experimental Department of Institute of Obstetrics and Gynecology, The Third Affiliated Hospital of Guangzhou Medical University, Guangzhou, 510150, China

**Keywords:** apoptosis, cell cycle, cell proliferation, glucose, human chorionic trophoblast

## Abstract

This study investigated the effects of glucose and osmotic pressure on the proliferation and cell cycle of trophoblast cells. HTR8/SVneo cells were treated with 0 (no glucose), 1 (low glucose), 5 (normal), and 25 mmol/L (high glucose) glucose. In addition, the cells were treated with 5 mmol/L glucose (normal) and 5 mmol/L glucose + 20 mmol/L mannitol (mannitol). The cell morphology and proliferation were determined by microscopy and a cell counting kit-8 assay. The cell cycle and apoptosis were examined by flow cytometry. The cell number was relatively decreased and morphological changes were intermediate in the high-glucose group compared with the low-glucose groups. The proportion of cells in the G2/M phase was higher in the low-glucose group than in the other groups, and it was lower in the G1 phase and higher in the S phase in the high-glucose group than in the other groups. Compared with 24 h, cell proliferative activity was restored to a certain extent after 48 h in the high-glucose group. In summary, the blood glucose concentration might influence the proliferation of trophoblast cells. A high-glucose environment inhibited initial cell proliferation, which could be moderately restored after self-regulation. Furthermore, the proliferation of trophoblasts was not affected by the osmotic pressure.

## 1 Introduction

The placenta contains both maternal and embryonic tissues. Embryonic tissues mainly include fetal blood vessels and trophoblast cells. The latter are in direct contact with the maternal blood during pregnancy. The function of trophoblast cells can be affected by maternal blood glucose fluctuations, with various subsequent pathophysiological outcomes. In women with diabetes in pregnancy (DIP), the trophoblast cells may undergo a series of pathological changes in response to different blood glucose environments. A previous study has reported an increased placenta–fetal weight ratio with a rising placental volume and weight due to poor blood glucose control in women with DIP [1]. This might be caused by the release of vascular endothelial growth factor and fibroblast growth factor from the placenta [2].

In DIP, changes in placental morphology and histology may result in functional changes [3]. In our early clinical studies, it was found that the local villi of placental specimens from DIP women were immature, showing signs of edema and degeneration. The gap between the villi was narrow, with the evidence of chronic inflammation. These pathological changes can affect the exchange of substances and gases between the mother and fetus, which might be the cause of adverse pregnancy outcomes, such as stillbirth [4,5]. The relationship between the placenta and the fetus is also regulated by the immune system. In fact, many innate immune cells play an important role in the balance and equilibrium of the normal response during pregnancy. Moreover, the pathological states can disrupt certain cell populations and their cytokine mediators [6].

Prolonged exposure to a high-glucose environment can lead to systemic arterial disease, with an increased risk of trophoblast invasion failure and remodeling of the uterine spiral artery. The spiral arteries can still retain their contractile function and cause excessive blood flow into the intervillous space. This may result in increased osmotic and oxygen partial pressures in the embryo and placenta, which is similar to the pathogenesis of preeclampsia. The high osmotic and oxygen partial pressures can affect the proliferation and differentiation of trophoblast cells as well as the capacity of the terminal villous vascular bed, which ultimately causes spontaneous abortion and stillbirth [7].

The present study investigated the changes in the trophoblast cell proliferation and functions under various glucose concentrations and osmotic pressures. The results of this study will provide experimental evidence for the prevention and treatment of stillbirth in women with DIP.

## 2 Materials and methods

### 2.1 Cell line, reagents, and equipment

The human chorionic trophoblast cell line HTR8/SVneo was obtained from the American Type Culture Collection (Manassas, VA, USA) and preserved in our laboratory. Reagents and equipment included fetal bovine serum (FBS; Gibco, USA), dimethyl sulfoxide (Sigma, USA), RPMI 1640 medium (HyClone, USA), RPMI 1640 medium, no glucose (Thermo, Germany), pancreatin (Gibco, USA), penicillin–streptomycin solution (HyClone, USA), d-mannitol (Solarbio, USA), Cell Cycle Detection Kit (Shanghai Bestbio Company), phosphate-buffered saline (PBS; HyClone, USA), glucose powder (Hefei Bomei Biotechnology Co., Ltd.), Cell Cycle Detection Kit (Sigma Company, USA), Cell Apoptosis Detection Kit (Thermo, Germany), Cell Counting Kit-8 (CCK-8; Beyotime Biotechnology, Shanghai, China), absolute ethanol (Sinopharm Group Co., Ltd., Beijing, China), adjustable pipette (2.5–1,000 μL), desktop refrigerated ultracentrifuge (5424R), high-speed mini centrifuge (Eppendorf, Germany), Vortex oscillator (Vortex-Genie^®^, Science Industries, Inc.), CO_2_ incubator, −80°C refrigerator, thermostatic water bath (Thermo, Germany), electronic platform scale (Sartorius, Germany), laminar flow bench (Suzhou Antai Airtech Co., Ltd.), inverted microscope (Olympus, Japan), microplate reader (Promega, USA), pure water system (Millipore, USA), and flow cytometer (BD FACSAria™ III (BD FACS AriaIII/BD, USA).

### 2.2 Experimental groups

#### 2.2.1 Effects of different glucose concentrations on HTR8/SVneo cells

Based on the human blood glucose concentrations (normal, 3.9–6.1 mmol/L; diabetic ketoacidosis, 16.7–33.3 mmol/L or even higher; and hypoglycemia, fasting blood glucose <2.8 mmol/L or blood glucose level ≤3.9 mmol/L) [8,9], the experimental groups were as follows: no glucose, 0 mmol/L glucose; low glucose, 1 mmol/L glucose; high glucose (diabetic ketosis), 25 mmol/L glucose; and normal blood glucose, 5 mmol/L glucose.

#### 2.2.2 Effects of osmotic pressure on HTR8/SVneo cells

The experimental groups to assess the effects of osmotic pressure were as follows: normal, 5 mmol/L glucose; osmotic pressure control, 5 mmol/L glucose + 20 mmol/L mannitol.

### 2.3 Cell culture

HTR8/Svneo cells were cultured in 6-well plates containing RPMI-1640 complete medium with 10% FBS. When the growth density reached 50–60% confluency, the RPMI-1640 medium was discarded. The cells were washed two times with PBS, and then, the appropriate concentration of glucose was added, 2 mL per well, until the final concentration reached the one specified for each experimental group. The cells were labeled and incubated at 37°C for 24 h before they were harvested, recovered, passaged, and cryopreserved.

### 2.4 Cell proliferation detected by CCK-8

Cells in the logarithmic phase of growth were collected. Approximately 4 × 10^3^ cells were added into each well of a 96-well plate, cultured in RPMI-1640 complete medium, and incubated overnight in a 37°C incubator containing 5% CO_2_. After the cells adhered to the wall, the medium was aspirated, and the cells were washed with PBS twice. The primary solution (100 μL/well) was added according to the specifications for each group. The cells were placed in a 37°C incubator containing 5% CO_2_ for 24 h. Then, 10 μL of CCK-8 solution was added to each well. The cells were incubated in the dark for 1 h. The optical density was measured at 450 nm. The results of the experiment were based on 0 mmol/L blood glucose as the reference (set as 100%) to compare the proliferation rates of the other groups.

### 2.5 Cell cycle analysis by flow cytometry

When the cell density in the 6-well plate reached 80%, the medium was aspirated, and the residual medium was washed off with PBS. An appropriate number of cells and 0.25% trypsin were added to a culture flask, and the mixture was incubated at 37°C for 2–5 min. When the cytoplasm shrank and the intercellular space increased, an equal amount of RPMI-1640 complete medium with 10% FBS was used to terminate the digestion. The cells on the wall of the flask were gently blown repeatedly to separate them from the wall, thus forming a cell suspension. The cells were aspirated into a 1.5-mL Eppendorf tube and centrifuged at 1,000 rpm for 5 min. The cells were washed twice with PBS, centrifuged at 1,000 rpm for 5 min, and collected.

For cell fixation, the cells were resuspended in PBS and centrifuged at 1,000 rpm for 5 min. Next, 3–5 × 10^5^ cells were resuspended in 0.2 mL of PBS to form a single-cell suspension and then fixed by 2 mL of 70% ice-cold ethanol, mixed, sealed, and stored in a refrigerator at 4°C overnight.

For propidium iodide (PI) staining and detection, the single-cell suspension was fixed overnight and centrifuged at 1,000 rpm for 5 min. After removing the supernatant, the pellet was resuspended in 2 mL of PBS, mixed well, and centrifuged at 1,000 rpm for 5 min. The supernatant was discarded, and 50 μL of RNase and 450 μL of PI staining solution were added to the pellet. Detection of PI by flow cytometry was performed within 4 h, as shown previously [10], with the cell suspension carefully observed beforehand. If the cell suspension was poorly dispersed or clotting was observed, it was filtered with a 200-mesh or 50-μm-diameter nylon mesh before the detection by the flow cytometry.

### 2.6 Cell apoptosis detection by flow cytometry

When the cell density in the six-well plate reached 80%, the medium was aspirated, and the residual medium was washed off with PBS. An appropriate number of cells and 0.25% trypsin were placed into a culture flask and incubated at 37°C for 2–5 min. When the cytoplasm shrank and the intercellular space increased, an equal amount of RPMI 1640 complete medium containing 10% FBS was used to terminate the digestion. The cells on the wall of the flask were gently blown repeatedly to separate them from the wall, thus forming a cell suspension. The cells were aspirated into a 1.5 mL Eppendorf tube and centrifuged at 1,000 rpm for 5 min. The supernatant was discarded, and 195 μL of Annexin V-FITC binding solution was used to gently resuspend the cells. Approximately 5 × 10^4^ to 1 × 10^5^ resuspended cells were obtained and centrifuged at 1,000 rpm for 5 min. Then, 5 μL of Annexin V-FITC was added to the cell suspension, which was gently mixed and placed in an incubator in the dark at room temperature (0–25°C) for 10 min. Next, the cells were centrifuged at 1,000 rpm for 5 min. The supernatant was discarded, and 190 μL of Annexin V-FITC binding solution was used to resuspend the cells. Then, 10 μL of PI staining solution was added, and the cell suspension was gently mixed and cooled in an ice bucket in the dark. Apoptosis was detected by flow cytometry, as described previously [11]. Annexin V-FITC staining appeared as green fluorescence, while PI staining appeared as red fluorescence.

### 2.7 Statistical analysis

Each experiment was repeated three times. All statistical analyses were performed by using SPSS 22.0 (SPSS Inc., IBM, New York, NY, USA). GraphPad 6 software (Software Inc., La Jolla, CA, USA) was used for making graphs. Categorical data were presented as a percentage or frequency. The differences between groups were compared by using Fisher’s exact test or the *c* ^2^ test. Continuous data were first tested for normal distribution. Normally distributed data were presented as the mean ± standard deviation. Non-normally distributed data were presented as the median and interquartile range. If the two sets of samples conformed to normal distribution, the independent-samples *t* test was used. Otherwise, the nonparametric Wilcoxon rank sum test was applied for comparison.

## 3 Results

### 3.1 Effects of different glucose concentrations on the morphology and proliferation of HTR8/SVneo cells

**Figure 1:**
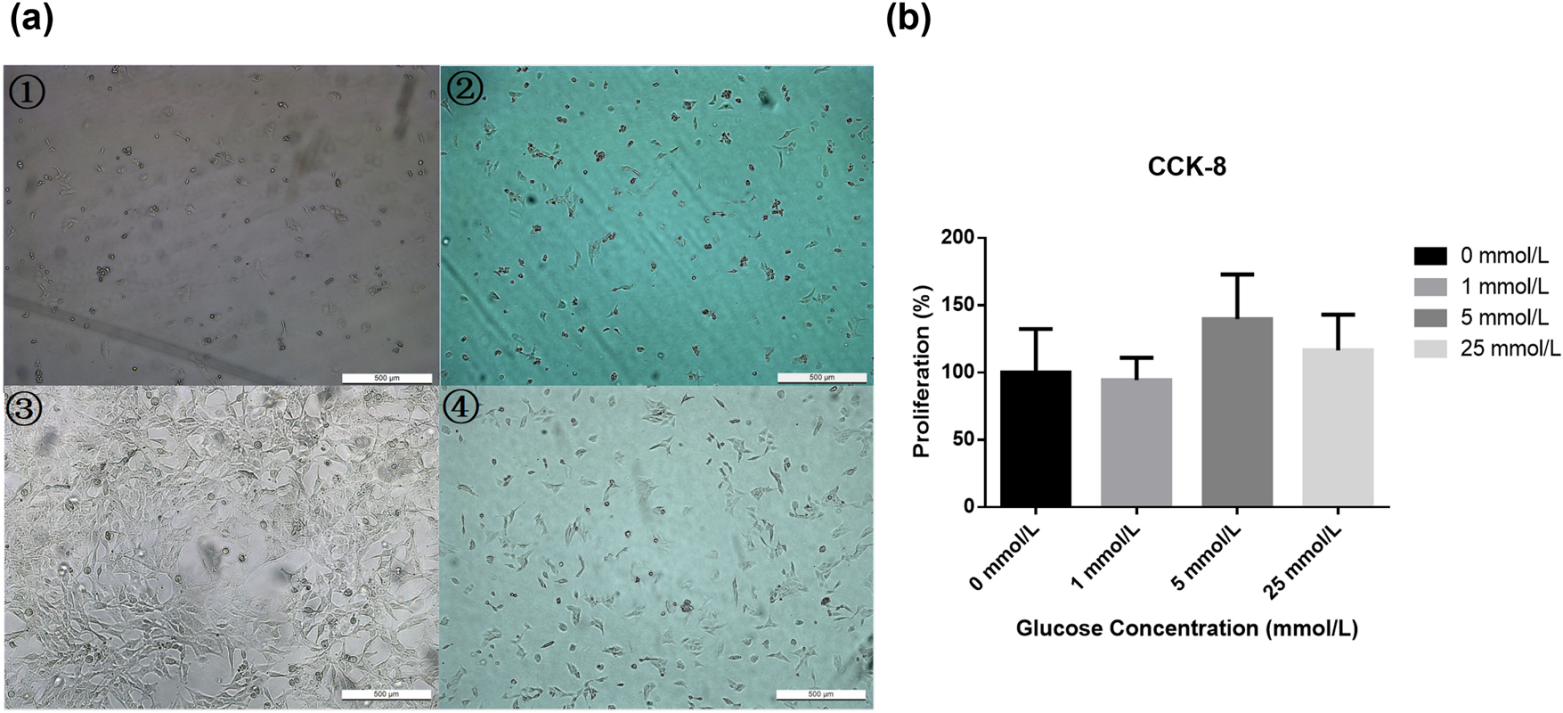
(a) After culturing for 24 h, the morphological changes in cells were observed under a light microscope (magnification, 200×). The cells in the normal group had a round and full cytoplasm, polygonal adherent growth, and an increased cell number. The cells in the no-glucose group had similar characteristics to the low-glucose group, with fewer adherent cells, shrunken cell morphology, and increased cell debris. The number of cells in the high-glucose group was relatively reduced, without obvious cell debris. The morphological changes were intermediate between the low-glucose group and the normal group. (b) The cell proliferation rate was detected by the CCK-8 assay. The cell proliferation rate of the no-glucose group was similar to that of the low-glucose group. The cell proliferation rate was the highest in the normal group. The proliferation rates of the other groups were decreased to various degrees. Note: ① 0 mmol/L, no-glucose group; ② 1 mmol/L, low-glucose group; ③ 5 mmol/L, normal group; ④ 25 mmol/L, high-glucose group.

After culturing the cells for 24 h, the changes in cell morphology were observed under an optical inverted microscope. The cells in the normal group had a round and full cytoplasm, polygonal adherent growth, and an increased cell number. The cells in the no-glucose group had similar characteristics to those of the low-glucose group: there were fewer adherent cells, the cells had a shrunken cell morphology, and there was increased cell debris. In the high-glucose group, the number of cells was relatively reduced, and no obvious cell debris was detected. The morphological changes observed were intermediate between those of the low-glucose group and the normal group (Figure 1a).

The cell proliferation rate of the no-glucose group was similar to that of the low-glucose group, while the cell proliferation rate was the highest in the normal group. The proliferation rates of the other groups were decreased to various degrees, although the differences were not statistically significant (*P* = 0.385) (Figure 1b). These results suggest that the glucose concentration might have some influence on the proliferation of trophoblast cells.

### 3.2 Effects of different glucose concentrations on the cell cycle of HTR8/SVneo cells

**Figure 2:**
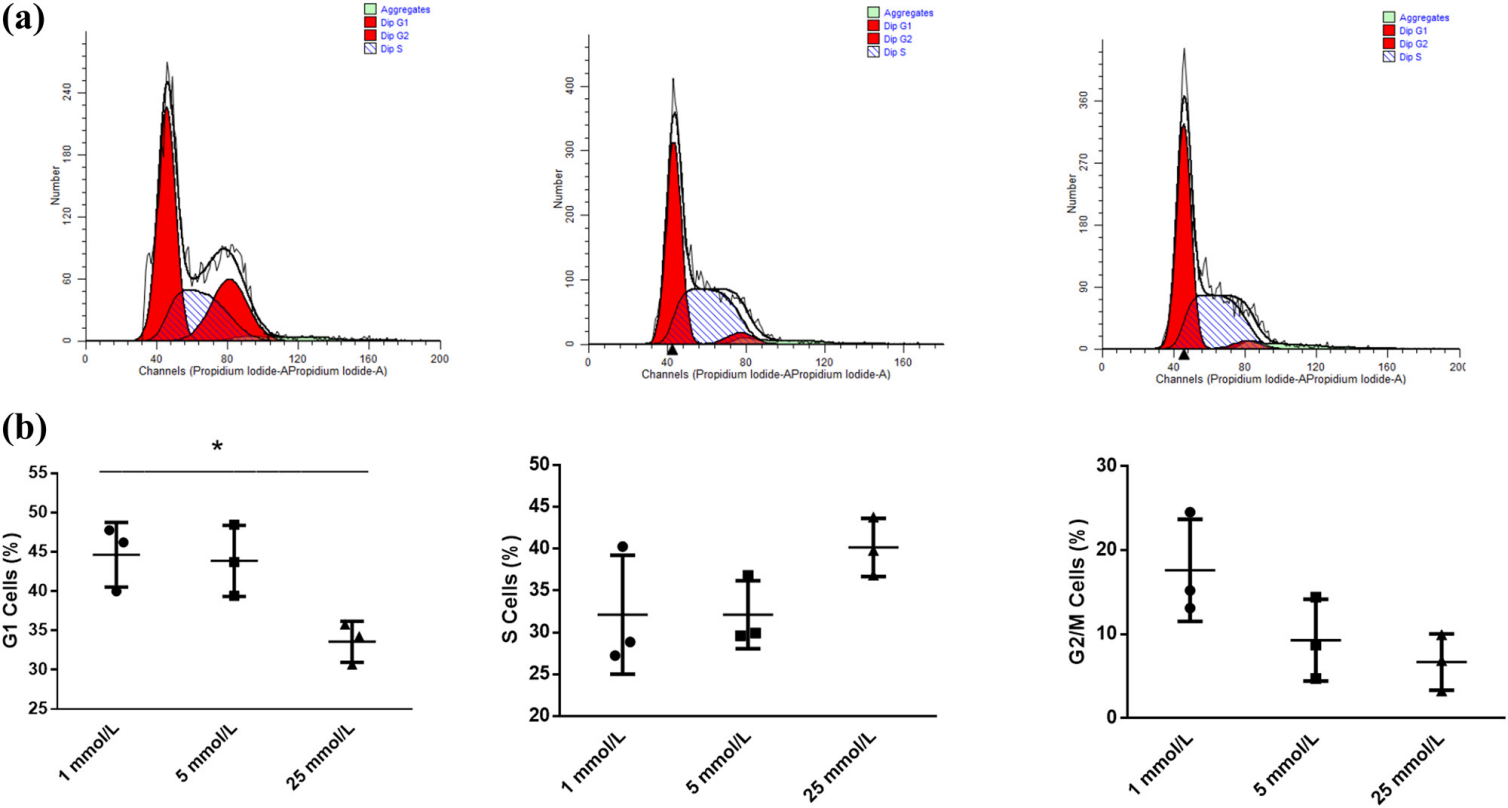
(a) Cell cycle analysis by flow cytometry. The proportion of cells was higher in the G2/M phase in the low-glucose group than in the normal group and the high-glucose group. The proportion of cells in the G1 phase was lower, while in the S phase was higher, in the high-glucose group compared with the other groups. (b) The proportions of cells in each group. The proportion of cells in the G1 phase in the high-glucose group was significantly decreased, while no significant changes were observed among the other groups. *P < 0.05. Note: 1 mmol/L, low-glucose group; 5 mmol/L, normal group; 25 mmol/L, high-glucose group.

As the above results suggest that the glucose concentration might affect the proliferation of trophoblast cells, we proposed that changing the glucose concentration might affect the normal cell cycle. Since the no-glucose group and the low-glucose group had a similar proliferation rate and cell morphology, we only tested the low-glucose group. The PI flow cytometry method was used to evaluate the cell cycle of trophoblasts in each group. The results showed that the cell proportion was higher in the G2/M phase in the low-glucose group than in the normal group and the high-glucose group. In addition, the proportion of cells in the G1 phase was lower while that in the S phase was higher in the high-glucose group, compared to the other groups (Figure 2a). The proportion of cells in the G1 phase in the high-glucose group was significantly decreased (*P* < 0.05), while there were no significant changes between the other groups (*P* = 0.279) (Figure 2b).

### 3.3 Effects of different glucose concentrations on the apoptosis of HTR8/SVneo cells

**Figure 3:**
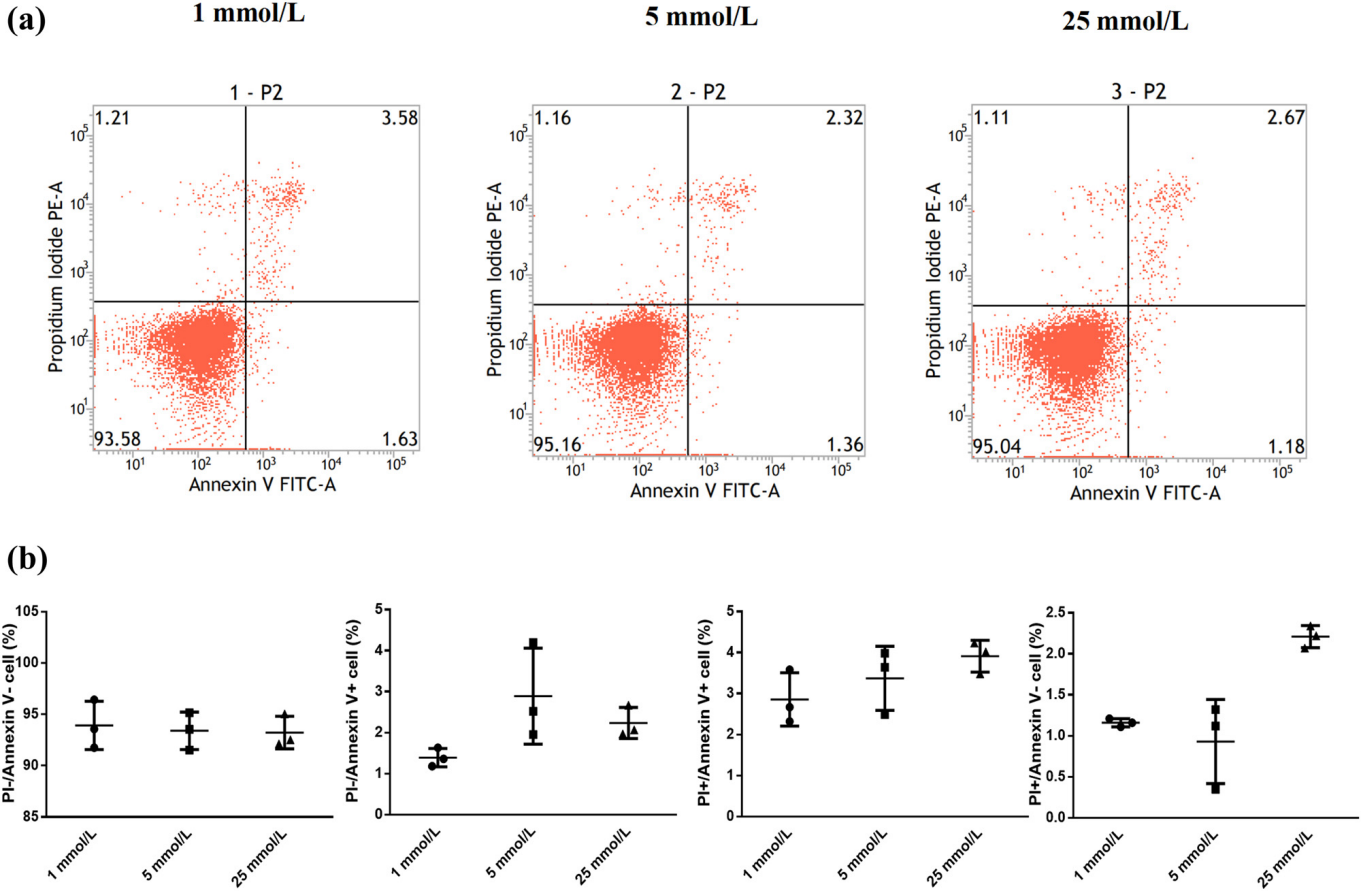
(a) Detection of cell apoptosis by flow cytometry. (b) Apoptosis in each group. The occurrence of apoptosis showed no significant difference among groups. Note: 1 mmol/L, low-glucose group; 5 mmol/L, normal group; 25 mmol/L, high-glucose group. FITC, fluorescein isothiocyanate.

Flow cytometry detection of Annexin V and PI double staining was applied to measure the apoptosis rate in each treatment group. There was no statistically significant difference in the occurrence of apoptosis among groups (*P* = 0.119) (Figure 3).

### 3.4 Comparison of HTR8/SVneo cells after different incubation periods under different glucose concentrations

**Figure 4:**
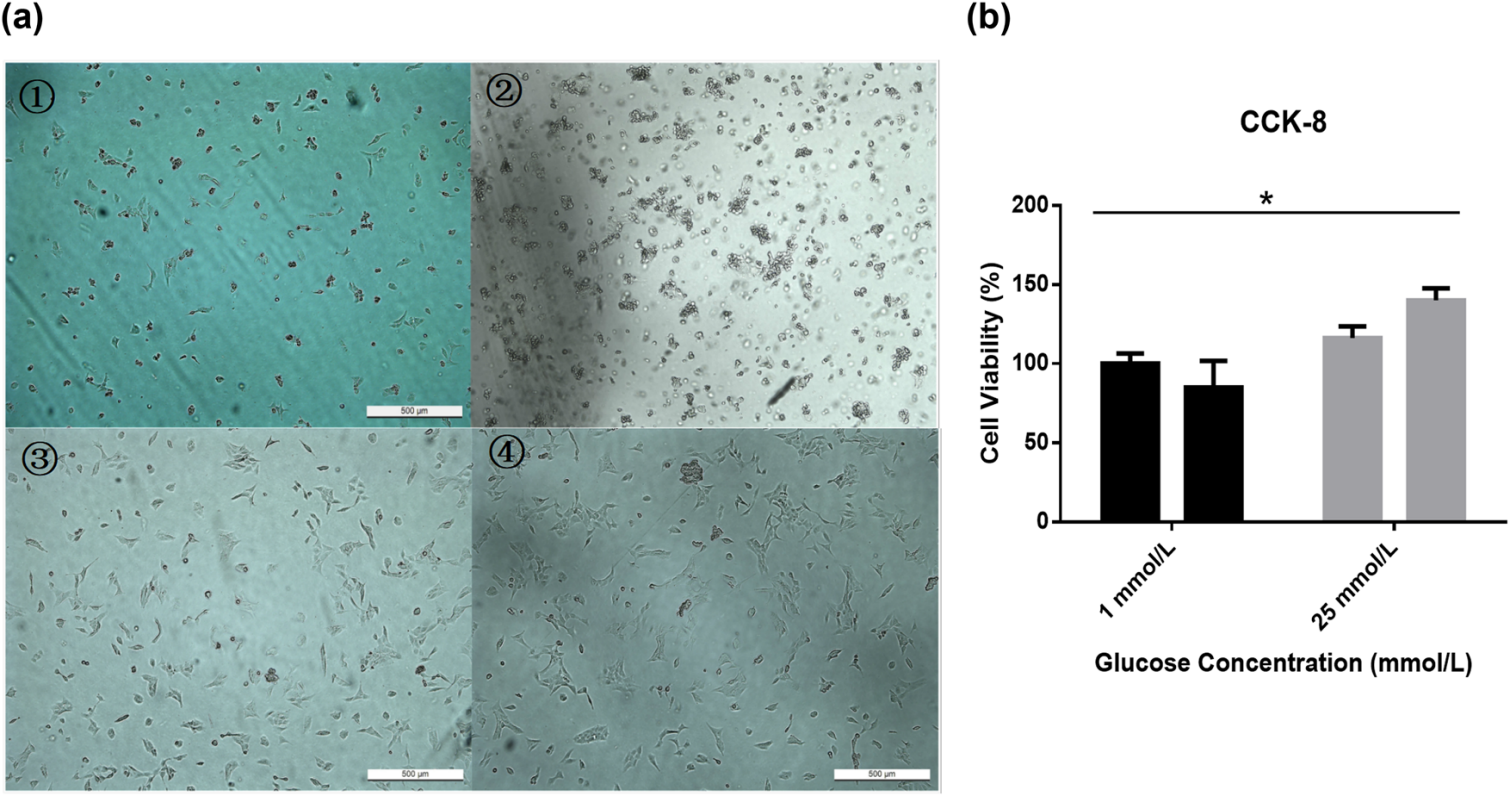
(a) The morphological changes of cells treated with 1 or 25 mmol/L glucose at 24 or 48 h were observed under a light microscope (magnification, 200×). (b) The cell viability of cells treated with 1 or 25 mmol/L glucose detected at 24 or 48 h. Compared with the proliferative activity at 24 h, the proliferative activity was restored to a certain extent after 48 h of culture in the high-glucose group. The proliferative activity continued to decrease in the low-glucose group. ① 1 mmol/L (24 h); ② 1 mmol/L (48 h); ③ 25 mmol/L (24 h); and ④ 25 mmol/L (48 h). *P < 0.05.

The morphological changes in the cells treated with various glucose concentrations were observed under an inverted optical microscope at 24 and 48 h. The results showed that, compared with 24 h, the proliferative activity was restored to a certain extent after 48 h of culture in the high-glucose group, while the proliferative activity continued to decrease in the low-glucose group (*P* < 0.05) (Figure 4).

### 3.5 Effects of osmotic pressure on HTR8/SVneo cells

**Figure 5:**
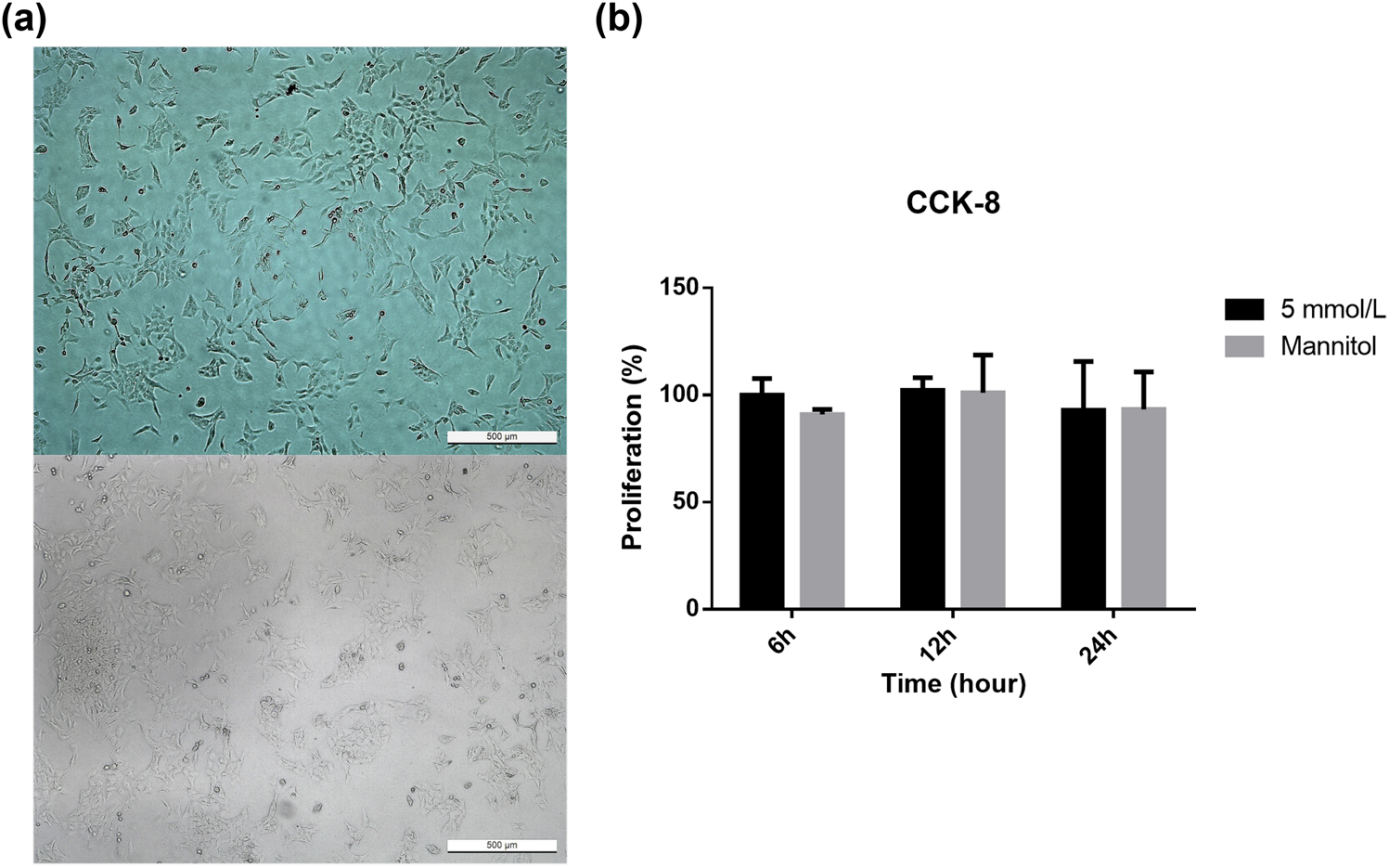
(a) After culturing for 24 h, the morphological changes in trophoblast cells treated with or without mannitol were observed under a light microscope (200×) to assess the effects of osmotic pressure. The number of trophoblast HTR8/SVneo cells was relatively increased in the mannitol-treated cells. The cells were round and had a full cytoplasm, with polygonal adherent growth and little differences in density. (b) The cell viability of these cells was detected at different time points. No significant differences in cell proliferation were observed among groups. Note: ① 5 mmol/L = 5 mmol/L glucose, normal group; ② mannitol = 5 mmol/L glucose + 20 mmol/L mannitol, osmotic pressure control group.

The changes in the morphology of cells treated with mannitol were observed under an inverted optical microscope. The number of trophoblast HTR8/SVneo cells was relatively increased in the mannitol-treated cells. The cells appeared round and had a full cytoplasm, with polygonal adherent growth and little differences in density (Figure 5a).

After 6, 12, and 24 h, the proliferative activity was detected by the CCK-8 assay. There were no statistically significant differences in the cell proliferation between groups (*P* = 0.237) (Figure 5b). These results suggest that the proliferative activity of the trophoblast cells was not affected by the osmotic pressure.

## 4 Discussion

Recent clinical studies have shown that DIP is associated with poor maternal–fetal outcomes, but the mechanisms are unclear [12]. Cell proliferation and division are the most basic and essential life processes in all living organisms. The cycle of cell growth and division is called the cell cycle, which refers to the whole process from the completion of one division to the end of the next division. It is divided into four phases [13,14,15]. The checkpoints in each phase are regulated by related cyclins and cytokines that control cell proliferation and division. The proliferation of cells is inhibited by the restriction point [5]. It is also possible that the cells accelerate through the restriction points of the G1–S transition, resulting in a decreased number of cells in the G1 phase. Retinoblastoma protein (pRb) is a negative regulator of the G1 restriction point. The accumulation of phosphorylated pRb and the E2F-pocket protein complex can inhibit cellular DNA synthesis and cause cells to exit the cell cycle, with a subsequent halt in the cell proliferation [16,17]. In the present study, we demonstrated that the proportion of cells in the G2/M phase was higher in the low-glucose group than in the normal and high-glucose groups. In addition, the proportion of cells in the G1 phase was lower while that in the S phase was higher in the high-glucose group compared with the other groups.

Meanwhile, the glucose concentration had no effect on cell apoptosis. A high-glucose environment inhibited cell proliferation at the initial stage, but it could be restored after self-regulation. A low-glucose environment decreased the cell proliferation. Our results also indicated that the osmotic pressure had no influence on the morphology or proliferative activity of trophoblast cells. Moreover, we found that decreased cyclin A/CDK2 activity could inhibit the transformation from the S phase to the G2/M phase, resulting in reduced cell proliferation. Furthermore, our study showed that the proportion of cells in the G2/M phase was higher in the low-glucose group than in the normal group and the high-glucose group. In addition, the proportion of cells in the G1 phase was lower while that in the S phase was higher in the high-glucose group compared to the other groups. These findings are consistent with the results reported previously. However, the proliferative activity of cells in the high-glucose group was restored to a certain extent after culturing for 48 h compared with 24 h, while a decreased proliferative activity persisted even after 48 h in the low-glucose group. This finding suggests that the effect of a low-glucose concentration on the proliferation of human trophoblast cells might be more significant and earlier than that of a high-glucose concentration.

Hinck et al. found that the rat trophoblast cell line Rcho-1 had an impaired endoreduplication process under high-glucose conditions [18]. However, it also has been reported that the response of trophoblast stem cells to glucose concentration changes is cell line dependent [19]. In addition, it has been shown that a high-glucose concentration can induce cell proliferation in cancer cells [20].

Apoptosis is a type of programmed cell death. It is a process often characterized by specific morphological changes and energy-dependent biochemical alterations. Apoptosis is considered to be an important part of various cellular processes, including normal cell renewal, immune system development and onset, hormone-dependent atrophy, embryonic development, and chemically induced cell death [21]. In diabetic pregnant rat models, it has been demonstrated that a high-glucose level can cause the placenta to have vasodilatation, an increased vascular wall membrane thickness, trophoblast proliferation, villous edema, accumulation of glycogen in various layers of the placenta, varying degrees of dilated endoplasmic reticulum, mitochondria degeneration, increased oxidative stress, and activated apoptosis of the placental tissue [22].

Moreover, Ji et al. have reported that trophoblasts induced by a high-glucose concentration have a low level of miR-193b expression, resulting in a high rate of apoptosis [23]. However, a study by Magee et al. has suggested that women with gestational diabetes have an increased risk for macrosomic newborns, while larger placentas are often accompanied by decreased apoptosis [24]. Our study found no obvious differences in apoptosis between the low- or high-glucose environment and the normal glucose environment.

Furthermore, necrosis and necroptosis can be initiated by mediators and proinflammatory cytokines such as interleukin-6 and tumor necrosis factor, with subsequent damage to the cell membrane. At the same time, the release of lactate dehydrogenases might amplify reactive oxygen species (ROS) in response to oxidative stimuli [25,26].

A high-glucose environment can increase the oxygen demand of trophoblasts, accelerate the synthesis of purine nucleotides, stimulate the decomposition of nucleotides, increase the concentration of nucleotide degradation products, and produce excessive ROS [27]. Excessive ROS can affect the function of trophoblast cells and their mitochondria through the opening of ion channels, lipid peroxidation, protein modification, and DNA oxidation, thus leading to trophoblast cell division arrest and apoptosis [28]. Huppertz et al. believe that a low-oxygen and low-ROS environment causes trophoblasts to have a higher proliferative activity and a lower invasion activity [28]. The premature increase of oxygen partial pressure may also reduce the proliferative ability of trophoblast cells [29].

ERK1/2 can simultaneously phosphorylate antiapoptotic molecules, such as BadSer112, and activate transcription factors to stimulate the expression of cell survival-related genes to provide antiapoptotic effects. In addition, Ozmen et al. have demonstrated in a rat model that abnormal placental and embryonic development caused by hyperglycemia may be related to the reduction of ERK1/2 phosphorylation [30].

Sánchez-Santos et al. have reported that trophoblast outgrowth induced by a high-glucose concentration is mediated by ROS [31]. Tao et al. also have found that a high-glucose treatment inhibits the viability, migration, and invasion of HTR8-S/Vneo cells by downregulating the expression of placental growth factor; increased ROS production was also detected in the high-glucose group [32]. Moreover, Peng et al. have shown that high-glucose levels can suppress the viability and proliferation of HTR8/SVneo trophoblast cells through the miR-137/PRKAA1/interleukin-6 axis [33]. Furthermore, a high-glucose concentration may cause significant changes in various functional pathways such as fatty acid β-oxidation, phospholipid metabolism, and phosphatidylinositol phosphate signaling [34].

It has been reported that hyperglycemia can affect trophoblast functions, early placentation, and pregnancy outcome [35]. In addition, Majali-Martinez et al. found that the cell viability of primary human first trimester trophoblasts at 7–8 weeks of gestation was unaffected by a high-glucose concentration (25 mM d-glucose), whereas pooled samples from 7 to 8 and 11 to 12 weeks of gestation showed a significant reduction in the number of viable cells, and samples from 9 to 10 weeks of gestation demonstrated the opposite trend. Consistent with the results of our study, the reduction in the number of viable cells could not be explained by an increased number of apoptotic cells, while downregulation of the G2/M to G1/G0 ratio under high-glucose treatment might also reflect changes in the cell cycle, resulting in the reduced number of viable cells [36]. Altogether, these results imply that maternal diabetes can alter placental development during the first trimester [37]. Our study found that the proliferation of trophoblasts could be inhibited in the early stage, without obvious apoptosis, in a high-glucose environment. The proliferative activity of the trophoblast cells continued to decrease after culturing for 48 h in a low-glucose environment.

Similarly, it is possible that the death of nerve cells in a low-glucose environment is caused not only by the lack of glucose supply but also by a reaction to the reintroduction of glucose after prolonged glucose deprivation [38]. In addition, Bhattacharya et al. have found that mammalian target of rapamycin signal transduction is decreased and that autophagy is increased in gastric cancer cells; although the proliferative activity is affected in these cells, apoptosis is not [39]. In the present study, we found that a low-glucose environment could inhibit the proliferation of trophoblast cells. The trophoblast cells died continuously even beyond 24 h. Based on the above findings, we predicted that an increased glucose concentration might lead to glucose reperfusion and induce the accelerated death of trophoblasts. Moreover, the similar rates of trophoblast apoptosis in a low-glucose environment compared to a normal environment might be related to the increased autophagy.

Telomeres are composed of repetitive DNA fragments that gradually shorten after each cell division. The regulation of telomere length is crucial to the development and survival of organisms. A short telomere can activate the DNA damage response and cause cell cycle arrest [40]. Whether a low-glucose environment affects the telomere length and telomerase function of trophoblasts requires further studies.

Based on the above research, we believe that a low-glucose environment can affect trophoblast proliferation. In a high-glucose environment, the cell proliferation was inhibited at the initial stage, but the cells could restore certain proliferative activity through self-regulation. In patients with DIP, the potential negative effects to trophoblast cells might be higher under hypoglycemic conditions than under hyperglycemic conditions.

Nevertheless, our study is not without limitations. Stillbirth occurs due to multiple pathogenic factors. Without cell function studies, the findings on the inhibitory effects of hypoglycemia on trophoblast cell activity are inadequate to clarify the pathophysiological mechanism in detail. Further animal experiments are needed to study the effects of a low-glucose environment on the function of trophoblast cells. Our study was performed with cell lines *in vitro*. Future research should be expanded to include studies using primary cells, pathological specimens, and animal models.

## 5 Conclusions

The blood glucose concentration might influence the proliferation of trophoblast cells. A high-glucose environment could reduce the number of trophoblast cells in the G1 phase. However, changing the glucose concentration had no significant effect on the early apoptosis of trophoblast cells. In addition, a low-glucose environment affected the proliferation of trophoblast cells, and a high-glucose environment inhibited the initial cell proliferation, but the cells could restore a certain proliferative activity after self-regulation. Finally, the proliferation of trophoblasts was not affected by the osmotic pressure.
